# Supplementary material for: Uptake of COVID-19 vaccine and associated factors among Health Care Workers at Queen Elizabeth Central Hospital in Malawi: A cross-section study
Source: PLOS Glob Public Health. 2024 Dec 5;4(12):e0003993. doi: 10.1371/journal.pgph.0003993 (PMC11620641; doi:10.1371/journal.pgph.0003993)
Supplement: S1 Questionnaire — (DOCX) [file pgph.0003993.s001.docx]

**Supplementary file**

**Participant Questionnaire**

**PARTICIPANT’S QUESTIONNAIRE**

**FACTORS ASSOCIATED WITH UPTAKE OF COVID-19 VACCINE AMONG HEALTH CARE WORKERS AT QECH IN BLANTYRE, MALAWI.**

Date…………………………………………………………………………………………

Participant’s identity number/code………………………………………….

Name of Research Assistant……………………………………………….

**Instructions**

Please tick in the box of an appropriate response and fill where appropriate.

**SOCIAL DEMOGRAPHIC INFORMATION**

1. Age………………………………………………………..
2. Gender
   1. Male
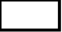

   2. Female
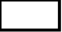

3. Marital status
   1. Married
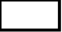

   2. Single
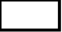

   3. Divorced/Widowed
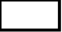

   4. Separation
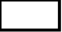

4. What is your religion
   1. Christian
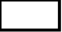

   2. Muslim
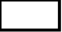

   3. Other
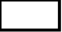

5. Where do you stay?……………………………………………………………
6. What is your profession?
   1. Clinical HCWs (doctors, clinicians, nurses, and physiotherapists)
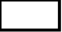

   2. Nonclinical HCWs (pharmacists, laboratory personnel, radiologists)
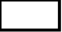

   3. Hospital attendant
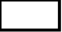

   4. Other
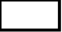

7. How long have you worked at QECH?
   1. Less than 1 year
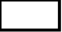

   2. 1year–3years
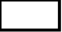

   3. 4years–7years
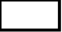

   4. 7years–11years
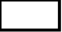

   5. 12 years and above
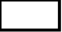

8. What is your highest level of education attained?
   1. No education attended
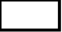

   2. PSLCE
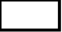

   3. JCE
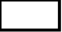

   4. MSCE
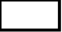

   5. Diploma
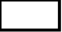

   6. Bachelor’s degree
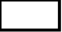

   7. Masters
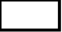

   8. PhD
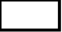

   9. Other
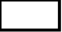

9. What is your family’s monthly income?
   1. Less than MK50000
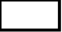


b) MK50000–MK100000
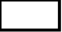


c) MK100000–MK200000
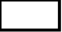


d) MK200000–MK300000
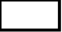


e) Above MK300000
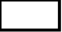


1. Other than work, do you have any sources of money?
   1. Yes
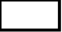

   2. No
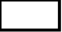

2. If yes, specify.…………………………………………………………………
3. How many children do you have?
   1. No child
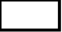

   2. 1
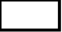


c) 2–4
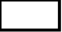


d) 5–7
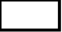


e) 8–11
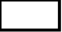


f) 12 and above
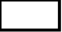


1. How many are you in your household?
   1. 1
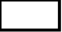


b) 2–4
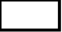


c) 5–7
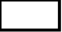


d) 8–11
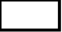


e) More than11
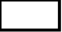


1. Who is your employer?
   1. Government
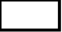

   2. NGO
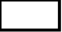

   3. Other
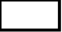

2. Which department are you working in?
   1. COVID-19 isolation department
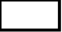

   2. Ear, nose and throat
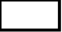

   3. High Dependency Respiratory Unit (HDRU)
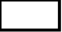

   4. Male or female medical (3Bor 4A)
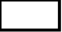

   5. Male or female surgical (5Aor5B)
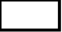

   6. Adult Emergency and Trauma Centre (AETC)
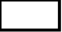

   7. Accidents and Emergency (Aand E)
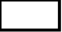

   8. Orthopaedic
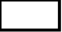

   9. Gynaecological
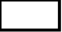

   10. Chatinkha nursery
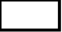

   11. PediatricMoyo
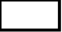

   12. Pediatric Special Care Ward (PSCW)
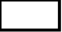

   13. Postnatal
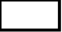

   14. Laboratory
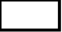

   15. Pharmacy
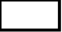

   16. Radiology
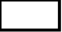

   17. Physiotherapy
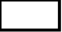

   18. Other
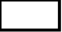

3. Are you on health insurance scheme?
   1. Yes
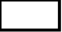

   2. No
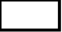


**PERSONAL MEDICAL HISTORY**

1. What is your smoking status?
   1. Current smoker
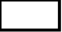

   2. Ex-smoker
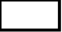

   3. Not a smoker
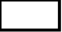

   4. Other
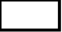

2. Do you have chronic illness?
   1. Yes
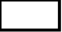

   2. No
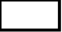

3. If yes, which medical condition do you have?
   1. Diabetes
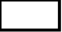

   2. Hypertension
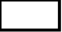

   3. Asthma
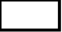

   4. Heart disease
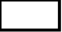

   5. Other
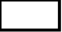

4. Have you ever refused any vaccination in the past?
   1. Yes
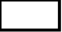

   2. No
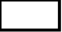

   3. Not sure
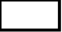


**COVID-19 AND PERSPECTIVES**

1. Were you trained or oriented about COVID-19?
   1. Yes
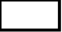

   2. No
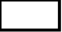

2. Do you know the name of COVID-19 vaccine being used in Malawi?
   1. Yes
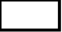

   2. No
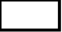

3. Have you ever worked in COVID-19 isolation unit?
   1. Yes
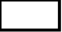

   2. No
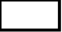

4. Do you think you have been exposed to or infected with COVID-19 without testing?
   1. Yes
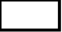

   2. No
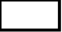

   3. Not sure
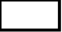

5. Have you ever tested positive for COVID-19?
   1. Yes
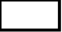

   2. No
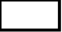

6. Has someone you know ever tested positive for COVID-19?
   1. Yes
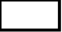

   2. No
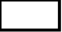

   3. Not sure
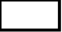

7. If yes, who tested positive for COVID-19?
   1. Family member
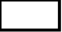

   2. Relative
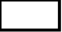

   3. Friend
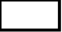

   4. Workmate
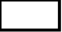

   5. Other
8. How do you perceive COVID-19?
   1. Very high
   2. High
   3. Medium
   4. Low
   5. Very low
   6. Not sure
9. Do you have a close relative or friend who died of COVID-19?
10. Yes
11. No
12. Not sure
13. Do you think the prevalence and death rate reports of COVID-19 by the Ministry of Health are real?
    1. Yes
    2. No
    3. Not sure
14. Are you at a higher risk of contracting COVID-19?
    1. Strongly agree
    2. Agree
    3. Neutral
    4. Disagree
    5. Strongly disagree

**COVID-19 VACCINE AND PERSPECTIVES**

1. Were you trained on the COVID-19 vaccine?
   1. Yes
   2. No
2. How did you know about COVID-19 vaccine?
   1. Newspaper, TV, Radio
   2. Social media (Facebook, WhatsApp, Twitter, Instagram, etc
   3. Internet
   4. Articles
   5. Church
   6. Family member
   7. Workmate
   8. Friend
   9. Other
3. In the past 3 months, have you received COVID-19 vaccine advertising messages?
4. Yes
5. No
6. Not sure

35. Which COVID-19 vaccine did you receive?

1. Johnson and Johnson
2. Pfizer
3. AstraZeneca
4. Other
5. None
6. If you received AstraZeneca, did you receive the first dose of AstraZeneca COVID-19 vaccine?
   1. Yes
   2. No
7. If yes, when?
   1. March 2021 – April 2021
   2. May 2021–June 2021
   3. July 2021
   4. Other

If you did not receive, proceed to question number 39

1. Where did you receive the first dose of COVID-19 vaccine?
   1. QECH
   2. Elsewhere
2. Did you receive the second dose of AstraZeneca COVID-19 vaccine?
   1. Yes
   2. No
3. If you received, when?
   1. March 2021 – April 2021
   2. May 2021–June 2021
   3. July 2021
   4. Other

If you did not receive, proceed to question number 44

1. Where did you receive the second dose of AstraZeneca COVID-19 vaccine?
   1. QECH
   2. Elsewhere
2. If you were vaccinated against COVID-19, did you experience any immediate side effects?
   1. Strongly agree
   2. Agree
   3. Neutral
   4. Disagree
   5. Strongly disagree
3. If you experienced side effects, which dose of AstraZeneca COVID-19 vaccine did you experience side effects?
   1. First dose
   2. Second dose
   3. First and second dose
   4. Other
4. If you did not receive or complete doses of COVID-19 vaccine, what were the reasons?
   1. Efficacy of COVID-19 vaccine
   2. COVID-19 vaccine immediate side effects
   3. COVID-19 vaccine unknown long term side effects
   4. Vaccine was out of stock
   5. Inadequate time at work
   6. Too much workload
   7. Long queues
   8. Didn’t know COVID-19 vaccination site
   9. Restricted by religion, culture, family member, etc
   10. Other
5. Do you think that COVID-19 vaccine may have long term side effects in future?
   1. Strongly agree
   2. Agree
   3. Neutral
   4. Strongly disagree
   5. Disagree
6. Do you think there is any harm or have concerns in being vaccinated against COVID-19?
   1. Strongly agree
   2. Agree
   3. Neutral
   4. Strongly disagree
   5. Disagree
7. Did you experience any inconveniences at vaccination site?
8. Yes
9. No
10. Not sure
11. Other than you, have some of your family members received COVID-19 vaccine?
    1. Yes
    2. No
    3. Not sure
12. Do you recommend your family members and friends to get vaccinated against COVID-19?
    1. Strongly agree
    2. Agree
    3. Neutral
    4. Disagree
    5. Strongly disagree
13. Disagree
14. Strongly disagree
15. Who do you trust most for information about COVID-19 vaccine?
    1. Newspaper, TV, Radio
    2. Social media (Facebook, WhatsApp, Twitter, Instagram, etc)
    3. Internet
    4. Articles
    5. Church
    6. Family member
    7. Work mate
    8. Friend
16. Other
