# Supplementary material for: Uptake of COVID-19 vaccine and associated factors among Health Care Workers at Queen Elizabeth Central Hospital in Malawi: A cross-section study
Source: PLOS Glob Public Health. 2024 Dec 5;4(12):e0003993. doi: 10.1371/journal.pgph.0003993 (PMC11620641; doi:10.1371/journal.pgph.0003993)
Supplement: Attachment — Submitted filename: Review Comments for PLOS Global Health article_20240930.pdf [file pgph.0003993.s005.pdf]

Results:

- Report results in the table and in-text to the same number of decimal places so that there is consistency throughout the document.
- **Table 2** – n=1 for ‘Other’ marital status. You have defined Other’ marital status as, separation, cohabitation and registered partnerships. Why not specify especially as only one person satisfied this criteria
- **Table 3** - Fix typographical errors (e.g. MedicalInsuranceScheme and Historyofvaccinerefusal)
