# Supplementary material for: Uptake of COVID-19 vaccine and associated factors among Health Care Workers at Queen Elizabeth Central Hospital in Malawi: A cross-section study
Source: PLOS Glob Public Health. 2024 Dec 5;4(12):e0003993. doi: 10.1371/journal.pgph.0003993 (PMC11620641; doi:10.1371/journal.pgph.0003993)
Supplement: Attachment — Submitted filename: Review Comments for PLOS Global Health article_20240331.docx [file pgph.0003993.s002.docx]

**Title:**

No comment.

**Abstract:**

No comment.

**Introduction:**

- SARS-CoV-2 stands for Severe acute respiratory syndrome coronavirus 2 and not Severe Acute Respiratory Syndrome (SARS-CoV-2) coronavirus.
- The sentence ‘Vaccination against SARS-CoV-2 promises to end the COVID-19 pandemic’ as the WHO declared the end of pandemic phase of COVID-19 in May 2023.
- Add the latest mortality data (as of April 2024) and not as of 17 August 2023.

**Method:**

- The study objectives are premised on HCW COVID-19 vaccine and associated factors. However, the population considered is of health professionals (1266) and not of HCWs (1710).
- Please recheck the minimum sample size calculation. Using a margin of error of 0.05 and a confidence interval of 95%, the minimum calculated sample size of a population of 1266 cannot be 222.
- More details of the random sampling technique should be provided.
- Clear inclusion and exclusion criteria need to be provided.
- Were study participants recruited from all wards/Departments? This is not made clear (although a list of wards/departments is given).
- How did you ensure validity and reliability of the research process and the data collected.

**Results:**

- Report results to the same number of decimal places.
- Report first on the overall vaccine uptake.
- The information provided in Table 1
- Table 2 needs to be reworked and reformatted.
- Table 2 – Add value for N
- Table 2 - What is ‘Other’ marital status?
- Table 2 – Segregate the age ranges further. Two categories is unusual and does not paint a full picture of the vaccine uptake by age range
- Table 2 – Segregate the professions further. E.g. Medical doctors, professional nurses, laboratory technicians, pharmacists, dentists, physiotherapists etc

Table 3 – Adjusted OR and p-values for some variables are missing

**Discussion:**

No comment.

**Study strengths and Limitations**

The statement, ‘The sample size was powered for high vaccine uptake, but as results show it is on the contrary hence this is a limitation that needs further investigation’, needs to be rephrased. It is not clear.

**Conclusion:**

No comment
