# Supplementary material for: Uptake of COVID-19 vaccine and associated factors among Health Care Workers at Queen Elizabeth Central Hospital in Malawi: A cross-section study
Source: PLOS Glob Public Health. 2024 Dec 5;4(12):e0003993. doi: 10.1371/journal.pgph.0003993 (PMC11620641; doi:10.1371/journal.pgph.0003993)
Supplement: Attachment — Submitted filename: RESPONSE TO REVIEWERS-PLOS GLOBAL PUBLIC HEALTH 2024.docx [file pgph.0003993.s004.docx]

**Title:**

No comment.

**Abstract:**

No comment.

**Introduction:**

- SARS-CoV-2 stands for Severe acute respiratory syndrome coronavirus 2 and not Severe Acute Respiratory Syndrome (SARS-CoV-2) coronavirus.

**Response**: The Severe acute respiratory syndrome coronavirus 2 (SARS-CoV-2).

- The sentence ‘Vaccination against SARS-CoV-2 promises to end the COVID-19 pandemic’ as the WHO declared the end of pandemic phase of COVID-19 in May 2023.

**Response:** Vaccination against SARS-CoV-2 contributed to the end of COVID-19 pandemic.

- Add the latest mortality data (as of April 2024) and not as of 17 August 2023.

**Response:** As of 13 April 2024, 704,753,890 had been infected with coronavirus and 7,010,681 died.

**Method:**

- The study objectives are premised on HCW COVID-19 vaccine and associated factors. However, the population considered is of health professionals (1266) and not of HCWs (1710).

**Response:** The sentence has been rephrased to read “HCWs”.

- Please recheck the minimum sample size calculation. Using a margin of error of 0.05 and a confidence interval of 95%, the minimum calculated sample size of a population of 1266 cannot be 222.

**Response:** The prevalence proportion of vaccine uptake from a previous study conducted in Malawi in March 2021 after the COVID-19 vaccination rollout had commenced was 82.5%. Using a margin of error of 0.05 and a confidence interval of 95%, the minimum calculated sample size was 190. We estimated that there would be an attrition/non-response rate of 10%. Accounting for non-response, the final sample size was 209.

- More details of the random sampling technique should be provided.

**Response:** The departmental In-charge was consulted, and participants were randomly selected from duty roster for enrolment.

- Clear inclusion and exclusion criteria need to be provided.

**Response:** The study inclusion criteria included HCWs with at least one year of working experience at QECH because, from the time of proposal writing, HCWs needed to be fully vaccinated against COVID-19, for at least a year, especially those who received a two-dose vaccine. The inclusion criteria had an age limit of 18 years and above since from the time of proposal writing the vaccines were not recommended for persons younger than 18 years. 18 years is the legal age of consent. The study excluded students since they were unlikely to have worked at QECH for one year at the time of proposal writing.

- Were study participants recruited from all wards/Departments? This is not made clear (although a list of wards/departments is given).

**Response:** The study participants were not recruited from all wards/departments, however, they were recruited from some selected wards/departments to present clinical, non-clinical and hospital attendants HCWs.

The study participants were recruited from the following wards/department: the COVID-19 isolation units, ENT, HDRU, Male and Female Medical (3B and 4A), Male and Female Surgical (5A and 5B), Accidents and Emergency (A and E), AETC, Orthopaedic, Gynaecological, Chatinkha Nursery, Paediatric Moyo, Paediatric Special Care Ward (PSCW), Postnatal, Laboratory, Pharmacy, Radiology and Physiotherapy.

- How did you ensure validity and reliability of the research process and the data collected.

**Response:** Data were collected using a questionnaire which was informed by the BeSD of the conceptual vaccination framework while following the research objectives. It was then compared to other data collection tools of similar studies on the COVID-19 vaccine acceptance and uptake conducted in Malawi and other countries. The data collection tools underwent expert review and were pretested to 10% of the calculated total sample size of a population not included in the study. Data were collected by the Principal Investigator (PI) to ensure the data’s completeness, timeliness and accuracy. Quality Checks (QCs) were conducted during data collection and entry.

**Results:**

- Report results to the same number of decimal places.

**Response:** Values in table have one decimal place except the p values.

- Report first on the overall vaccine uptake.

**Response:** From the 273 participants recruited in this study, 193 HCWs were fully vaccinated, representing 71% COVID-19 vaccine uptake.

- The information provided in Table 1

**Response:** This comment is not complete.

- Table 2 needs to be reworked and reformatted.

**Response:** The table has been reworked.

- Table 2 – Add value for N

**Response:** Value for has been added.

- Table 2 - What is ‘Other’ marital status?

**Response: ‘**Other’marital status means separation, cohabitation and registered partnerships. An asterix and the meaning have been added under the table.

- Table 2 – Segregate the age ranges further. Two categories is unusual and does not paint a full picture of the vaccine uptake by age range.

**Response:** To segregate the sample size of 273 into further age ranges, fitting that into a model, the outcome would be zeros, hence the model would be unbalanced. The age ranges in the study had some inherent limitations.

- Table 2 – Segregate the professions further. E.g. Medical doctors, professional nurses, laboratory technicians, pharmacists, dentists, physiotherapists etc.

**Response:** The above response applies to this. However, this may be considered as the study limitation, future studies may segregate the professions further.

- Table 3 –Adjusted OR and p-values for some variables are missing.

**Response:** The table has been reworked, it has covariates which were fitted in ‘likelihood-ratio test’, and have complete values.

**Discussion:**

No comment.

**Study strengths and Limitations**

The statement, ‘The sample size was powered for high vaccine uptake, but as results show it is on the contrary hence this is a limitation that needs further investigation’, needs to be rephrased. It is not clear.

**Response:** This limitation has been removed.

**Conclusion:**

No comment

**REVIEWER #2’S COMMENTS**

**Introduction**

In the introduction, it would be important if the source of the following data is recognized or cited; In Malawi, 1061 HCWs in the Blantyre district tested SARS-CoV-2 antibodies positive as of August 2023.

**Response**: In Malawi, according to Health Management Information System (HMIS) at Blantyre District Health Office (DHO), 1061 HCWs in the district tested SARS-CoV-2 antibodies positive as of August 2023.

**Discussion**

The author has put up a strong discussion, however i have noted that in the last statement of the second paragraph, the author is issuing what i believe to be a limitation, and recommendation, as demonstrated in the statement below (The study results should be generalized with caution since it was a single site study. We recommend a similar study in tertiary hospitals in central and northern region of Malawi). I believe the authors should focus on the discussion, and add such on the study limitations and recommendation.

**Response:** The statement has been added in the study limitation section **“**It was a single site study, hence study results should be generalized with caution. We recommend a similar study in tertiary hospitals in central and northern region of Malawi.”

With regards to the age brackets given, those less than 30 and those above thirty, even though advanced age is a risk factor severe COVID-19 disease, the authors need to clearly define which advanced age, as 30-40 is also considered relatively young, perhaps the authors can provide more details as how many were above the age of 40.

**Response:** According to this study, the age range was categorised into those less than 30 and those above 30 because to segregate the sample size of 273 into further age ranges, fitting that into a model, the outcome would be zeros, hence the model would be unbalanced. The age ranges in the study had some inherent limitations.
