# Supplementary material for: Uptake of COVID-19 vaccine and associated factors among Health Care Workers at Queen Elizabeth Central Hospital in Malawi: A cross-section study
Source: PLOS Glob Public Health. 2024 Dec 5;4(12):e0003993. doi: 10.1371/journal.pgph.0003993 (PMC11620641; doi:10.1371/journal.pgph.0003993)
Supplement: Attachment — Submitted filename: RESPONSE TO REVIEWERS.docx [file pgph.0003993.s006.docx]

**RESPONSE TO REVIEWERS**

**Results:**

• Report results in the table and in-text to the same number of decimal places so that there is consistency throughout the document.

**Response:** The results in the table and in-text have the same number of decimal places.

• Table 2 – n=1 for ‘Other’ marital status. You have defined Other’ marital status as, separation, cohabitation and registered partnerships. Why not specify especially as only one person satisfied this criteria.

**Response**: ‘Other status has been specified to ‘separation’.

• Table 3 - Fix typographical errors (e.g. MedicalInsuranceScheme and Historyofvaccinerefusal.

**Response:** The typographical errors have been fixed.
